# Respiratory rate - interobserver reliability study

**DOI:** 10.1186/1757-7241-23-S1-A14

**Published:** 2015-07-16

**Authors:** Jacob B Brodersen, Peter Hallas, Mikkel Brabrand

**Affiliations:** 1 Department of Medical Gastroenterology, OUH Odense University Hospital, Odense, Denmark; 2 Department of Emergency Medicine, Sydvestjysk Sygehus, Esbjerg, Denmark; 3 Copenhagen University Hospital, Department of Pediatric Anesthesia, Rigshospitalet, Copenhagen, Denmark; 4 Department of Emergency Medicine, OUH Odense University Hospital, Odense, Denmark

## Background

Measuring respiratory rate (RR) is one of the most basic clinical observations performed when accessing acutely ill patients. RR is included in most triage systems and risk stratifications tools, but unlike the other vital signs, RR is typically obtained by a manual count by the nursing staff. Considering how often RR is used in clinical practice and contained in triage systems, it is remarkable how few studies on inter-observer/rater agreement have actually been performed. Furthermore, the existing studies are all made with few observers and many patients, and none of them have been performed in an actual acute setting of an emergency department (ED). We therefore aimed to determine the interobserver variability of RR counts, using a larger number of observers on few same patients in the setting of an ED.

## Methods

This is a reliability study on the manual count of RR, based on video recordings made at the admission of acutely ill medical patients to the emergency department. The assessment of RR was done as a part of a larger triage study, making the raters unaware of the focus on RR. The main study was a cross-sectional study on the reliability of existing triage systems. The videos were recorded during the first 15 minutes after arrival of the patient to the Emergency Department at Sydvestjysk Sygehus, Esbjerg. The videos were anonymized and shown as part of the complete internet-based questionnaire. We calculated the individual Intra Class Coefficient.

## Results

Seven patient videos were assessed by eight observers. Each observer only assessed one case. All observers were trained nurses with a median experience of 15.23 years (range 0-37). The assessment of RR of each video ranged from 22-36, 24-32, 14-32, 12-30, 22-32, 20-30 and 19-30, respectively. The individual absolute ICC was 0.13 (95% confidence interval: 0.00-0.56).

## Conclusions

We found poor agreement comparing the actual number of breaths per minute and a very low ICC. However, our methods of using video recording in the ED on acutely ill patients as a base for inter-observer studies proved useful, and have potential for further use.

